# Influence of Body Composition and Muscle Power Performance on Multiple Frequency Speed of Kick Test in Taekwondo Athletes

**DOI:** 10.3390/sports12120322

**Published:** 2024-11-27

**Authors:** Gennaro Apollaro, Marco Panascì, Ibrahim Ouergui, Coral Falcó, Emerson Franchini, Piero Ruggeri, Emanuela Faelli

**Affiliations:** 1 Department of Neuroscience, Rehabilitation, Ophthalmology, Genetics and Maternal Child Health, University of Genoa, 16132 Genoa, Italy; gennaro.apollaro@edu.unige.it; 2 Centro Polifunzionale di Scienze Motorie, University of Genoa, 16132 Genoa, Italy; ruggeri@unige.it (P.R.); emanuela.faelli@unige.it (E.F.); 3 Department of Experimental Medicine, Section of Human Physiology, University of Genoa, 16132 Genoa, Italy; 4 High Institute of Sport and Physical Education of Kef, University of Jendouba, Kef 7100, Tunisia; brahim.ouerghi@issepkef.u-jendouba.tn; 5 Research Unit: Sport Sciences, Health and Movement, UR22JS01, University of Jendouba, Kef 7100, Tunisia; 6 Department of Sport, Food and Natural Sciences, Western Norway University of Applied Sciences, 5020 Bergen, Norway; coral.falco@hvl.no; 7 Martial Arts and Combat Sports Research Group, Sport Department, School of Physical Education and Sport, University of São Paulo, São Paulo 05508-030, Brazil; efranchini@usp.br

**Keywords:** combat sports, physical tests, anaerobic assessment, linear regression, Olympic sports

## Abstract

The Multiple Frequency Speed of Kick Test (FSKT_mult_) is used to investigate which characteristics are necessary for, contribute to, or limit the ability to repeat high-intensity intermittent efforts in taekwondo. This cross-sectional study investigated the relationship between anthropometric and body composition characteristics, muscle power performance, and sport-specific anaerobic performance. Nineteen black belt taekwondo athletes (mean ± SD age: 17.2 ± 2.4 years) volunteered to participate. Anthropometric and body composition characteristics (i.e., body height (BH), body mass (BM), fat mass (FM), body fat (BF%), and muscle mass (MM)) and physical performance (squat jump (SJ), countermovement jump (CMJ) tests, and FSKT_mult_) were assessed. Data were analyzed with correlation coefficients and simple linear regression. The statistical significance was set at *p* < 0.05. The total number of kicks in FSKT_mult_ (FSKT_total_) was significantly and positively correlated with MM (*r* = 0.521, *R*^2^ = 0.27, *p* < 0.05) and negatively with BF% (*r* = −0.499, *R*^2^ = 0.25, *p* < 0.05). The FSKT_total_ was significantly and positively correlated with SJ (*r* = 0.520, *R*^2^ = 0.27, *p* < 0.05) and CMJ (*r* = 0.508, *R*^2^ = 0.26, *p* < 0.05) performance. Body composition optimization, with appropriate physical training and dietary planning, is relevant in taekwondo as the improvement in the ability to repeat high-intensity intermittent efforts depends on MM, and its worsening on BF%. Lower limb muscle power positively influences the ability to repeat high-intensity intermittent efforts. Therefore, training programs should emphasize ballistic and plyometric exercises.

## 1. Introduction

In taekwondo, some sport-specific anaerobic testing protocols are studied and used more than others as they have methodological and application characteristics that better reproduce the physical and physiological demands of the intermittent high-intensity efforts of combat [1,2,3,4]. Aiming to improve the ecological validity over the initially designed continuous tests, the Multiple Frequency Speed of Kick Test (FSKT_mult_) is the first sport-specific test developed to assess the anaerobic capacity of taekwondo athletes in intermittent mode [1]. The FSKT_mult_ has a short protocol (five 10 s sets of all-out *bandal-chagi* execution alternating with 10 s of passive recovery) and features logical criterion and discriminant construct validity, sensitivity, and test–retest reliability [1,5]. Moreover, it is simple to conduct, non-invasive, requires low-cost instrumentation, and is easily implemented by coaches and strength and conditioning professionals in taekwondo gyms. Performance parameters derived (i.e., number of kicks in each set, total number of kicks, and kick decrement index) are easy to interpret as they are based on the number of valid kicks recorded by the evaluator [1]. In this regard, the inter-/intra-rater reliability of the FSKT_mult_ was also systematically investigated [1,5] as a basic criterion to support performance validity. A further sport-specific anaerobic test, the chest Taekwondo Anaerobic Intermittent Kick Test (TAIKT_chest_), although presenting similar methodological and executive characteristics to the FSKT_mult_ (six 5 s sets of all-out *bandal-chagi* execution alternating with 10 s of active recovery), expresses performance in terms of absolute and relative power (i.e., highest and mean power output of the sets of kicks, and fatigue index), as body mass is a criterion for categorizing taekwondo athletes in competitions [3,6]. In addition, valid kicks in the TAIKT_chest_ are automatically recorded by the electronic body protector eliminating the responsibility of the evaluator/s. It is crucial to highlight that the method of Tayech et al. [3], expressing TAIKT_chest_ performance in terms of power, has never been applied in the FSKT_mult_. Thus, this method could be easily used by increasing the number of parameters, information, and insights, for scientists and coaches, that can be obtained from the FSKT_mult_. In parallel, the higher cost of evaluation in the TAIKT_chest_, due to the use of electronic body protector that is not always available for all coaches, and the high inter-/intra-rater reliability of the FSKT_mult_ (intraclass correlation coefficient [ICC] ≥ 0.99) [1,5], could justify the greater use in practice of the latter.

The FSKT_mult_ is commonly used to investigate which characteristics are necessary for, contribute to, or limit the ability to repeat high-intensity intermittent efforts in taekwondo. This ability can be considered a determining factor for success in taekwondo competitions, as the short and dynamic actions, which occur in between skipping phases, determine the score [1,2,3,4]. In particular, the research focused on the relationship between anthropometric and body composition characteristics; lower limb muscle power, as assessed through heights achieved in the squat jump (SJ) and countermovement jump (CMJ) tests; and sport-specific anaerobic performance, obtained in the FSKT_mult_ [7,8,9]. The analysis of the influence of anthropometric and body composition characteristics on the ability to repeat intermittent high-intensity efforts in taekwondo is critical to find desirable or undesirable prerequisites for optimal anaerobic performance [9]. At the same time, typical kicking actions are characterized by high forces applied in short periods of time, requiring certain minimum power values to repeatedly successfully impact electronic scoring devices [6,10]. Therefore, the understanding of the influence of muscle power on the ability to repeat high-intensity intermittent efforts has a direct impact on training practices [7,8].

Santos et al. [8] analyzed the relationship of FSKT_mult_ performance with anthropometric and body composition characteristics, and SJ/CMJ performance. Only the fourth set of the FSKT_mult_ was significantly and negatively correlated with body height and fat mass (*r* = −0.51 and −0.61, respectively). In contrast, no significant correlation emerged between the FSKT_mult_ performance and that of the SJ and CMJ tests. Subsequently, Ojeda-Aravena et al. [9] studied the relationship of FSKT_mult_ and SJ/CMJ performance with body composition characteristics. Total number of kicks in the FSKT_mult_ was significantly and positively correlated with muscle mass (*r* = 0.56), while kick decrement index was significantly and positively correlated with fat mass and body mass index (*r* = 0.52 and 0.54, respectively). This study confirmed the absence of a relationship between the total number of kicks in the FSKT_mult_ and fat mass but showed a relationship with muscle mass that cannot be compared as a variable not previously considered [8]. Concurrently, the relationships found for the kick decrement index did not confirm the results of Santos et al. [8]. Interestingly, a clear pattern of relationship emerged between SJ/CMJ performance and body composition characteristics. In fact, both SJ and CMJ performance were significantly and positively correlated with muscle mass (*r* = 0.58) and negatively with fat mass, even when expressed as a percentage (*r* = −0.84–−0.89). Recently, Albuquerque et al. [7] determined the relationship between FSKT_mult_ and CMJ performance. In the study, the total number of kicks in the FSKT_mult_ was significantly and positively correlated with CMJ performance (*r* = 0.44) in disagreement with previous findings by Santos et al. [8].

Thus, the most recent data on the relationship between CMJ and FSKT_mult_ performance highlight the influence of muscle power on high-intensity repeated actions [7]. The latter aspect, when combined with the clear pattern of relationship between power performance and body composition characteristics [9], marks the importance of analyzing the relationship between these characteristics in parallel to better understand their influence on sport-specific anaerobic performance. Overall, the presented studies show a limited and contradictory framework on the topic [7,8,9]. Available information does not allow for us to delineate a pattern of relationship between anthropometric and body composition characteristics and FSKT_mult_. This is mainly caused by the fact that these studies did not analyze the same anthropometric [9] and body composition characteristics [8], or all the FSKT_mult_ performance [7,9], reducing the possibility of comparing the results. It is also important to add that in all studies [7,8,9], the performance parameters of the SJ, CMJ, and FSKT_mult_ were also not expressed in terms of absolute and relative power, thus limiting the insights that can be obtained from the relationships between these characteristics.

Therefore, the present study investigated the relationship between anthropometric and body composition characteristics, muscle power performance (SJ and CMJ), and sport-specific anaerobic performance (FSKT_mult_) in taekwondo athletes. Based on this, we also determined which anthropometric and body composition characteristics, and muscle power performance, could predict the main FSKT_mult_ performance. We hypothesized (a) the confirmation of the pattern of relationship between muscle power performance and body composition characteristics, (b) the presence of relationships between FSKT_mult_ performance and body composition characteristics (particularly fat mass and muscle mass), as well as (c) between FSKT_mult_ performance and muscle power performance.

## 2. Materials and Methods

### 2.1. Experimental Approach to the Problem

This cross-sectional observational study was conducted during the competitive season. In the week prior to experimentation, athletes conducted two familiarization sessions, with the testing procedures, to minimize the learning effect. The test sessions were conducted on two consecutive days. On the first day, anthropometric and body composition characteristics were assessed. After 24 h, the physical performance assessment session was performed, and the order of tests was established according to intensity: SJ test, CMJ test, and FSKT_mult_, respectively.

### 2.2. Participants

An a priori power analysis (G*Power 3.1.9.7; Heinrich Heine University in Düsseldorf, Germany) indicated that a total sample of 19 subjects would be required with the following settings, bivariate normal model test, two-tailed, power of 0.80, α-value of 0.05, and a correlation of *r* = 0.60, in accordance with previous studies [4,6]. Nineteen Italian taekwondo black belt athletes (9 males and 10 females; mean ± SD age: 17.2 ± 2.4 years; years of practice: 11.7 ± 2.2 years), from the same club, volunteered to participate in this study. Athletes were regularly engaged in national (i.e., national championships and cups) and international (i.e., G-1/E-1 competitions and/or European championships) competitions, and followed a standard training program of 8 weekly sessions (i.e., 6 taekwondo-specific training sessions and 2 strength training sessions), each lasting ~90 min. Athletes reported that they did not engage in any acute rapid weight loss strategies during the study period. At the time of recruitment, none of them had reported muscle or joint injuries during the past 6 months and none of them were taking drugs, medication, or dietary supplements, which could interfere with the experimental procedures. This study was approved by the Local Ethics Committee (University of Genoa. N. 2024/44), and all experimental procedures were conducted in accordance with the Declaration of Helsinki for human experimentations [11]. The athletes received instructions concerning the experimental design and any known risk, and they were also informed that they were free to withdraw from the experimental procedures at any stage. They signed an informed consent document to participate in this study, while for athletes under the age of 18, signed consent was obtained from their parents or guardian.

### 2.3. Procedures

**Figure 1:**
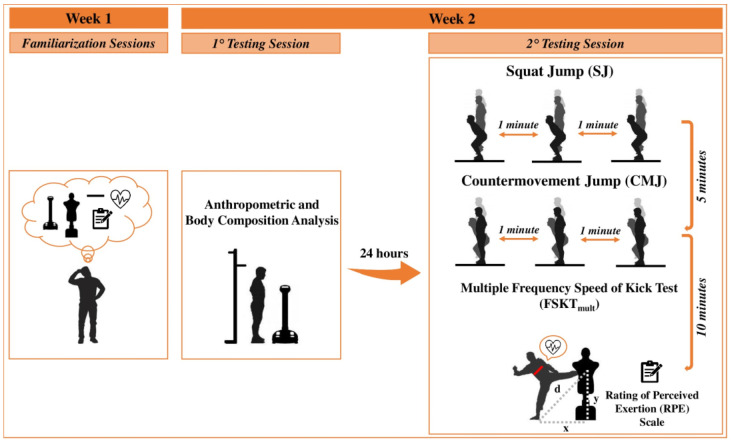
Schematic representation of the study design.

Both testing sessions were conducted by the same researcher at the athletes’ sports center, at the same time of day (10:00–12:00 a.m.) and under similar temperature (24–25 °C) and humidity conditions (48–51%) to avoid any diurnal variation in performance. In the 24 h before the two test sessions, athletes were asked to avoid consumption of caffeine and alcohol, as well as any strenuous physical activity. In addition, athletes performed the first and the second testing sessions without consuming food for 8 and 2 h beforehand, respectively. Prior to the second testing session, athletes performed a general and specific warm-up routine consisting of running, stretching, kicking, and punching at low intensity for a total of 15 min [1]. After 5 min of passive recovery, the athletes performed the tests. Three attempts were allowed for each muscle power test (SJ and CMJ), while only one attempt was allowed for the FSKT_mult_ [9]. A passive recovery interval of 1 min was applied between each attempt, while 5 or 10 min was given between each test to allow for complete recovery. Heart rate (HR) and rating of perceived exertion (RPE) were assessed during and after the FSKT_mult_, respectively. Athletes were instructed to give their best during physical tests. In addition, the same researcher who conducted the sessions consistently provided standard verbal encouragement to all athletes during testing. The experimental procedures are detailed in Figure 1.

### 2.4. Measures

#### 2.4.1. Anthropometric and Body Composition Analysis

Body height (BH) (cm) with athletes barefoot was measured with a stadiometer (*Seca Model 217; SECA GmbH & Co., KG., Hamburg, Germany*), with an accuracy of 0.1 cm. Then, body composition characteristics were analyzed using a bioelectrical impedance scale (*Tanita BC-420 MA; Tanita Corp., Tokyo, Japan*), with an accuracy of 0.1 kg. Following the manufacturer’s guidelines, athletes stepped on the bioelectrical impedance scale, wearing light athletic gear (shorts and t-shirt) and barefoot, and they stood still on the device platform while contacting the electrodes for ~30 s. Variables collected were body mass (BM) (kg), fat mass (FM) (kg), body fat (BF) (%), muscle mass (MM) (kg), and lean body mass (LMB) (kg). The body mass index (BMI) was calculated as BMI = BM/BH^2^ (kg·m^−2^).

#### 2.4.2. Muscle Power Performance

*Squat Jump (SJ) test*. Athletes performed the SJ on an electronic contact mat (*Globus Ergo Jump; Globus Inc., Codognè, Italy*), with an accuracy of 0.01 m, to determine the maximum height of the vertical jump. Athletes were asked to place their hands on their hips, feet well apart, adopt a flexed knee position (~90°) for 3 s, and then perform a vertical jump [9]. Three attempts were allowed with 1 min of passive recovery between them (within-session reliability: ICC = 0.991, *p* < 0.001; coefficient of variation [CV]: 3.3%).

*Countermovement Jump (CMJ) test*. Athletes performed the CMJ on the same electronic contact mat to determine the maximum height of the vertical jump. Athletes were asked to place their hands on their hips, with feet spread apart; then, they performed a downward motion (no limitation was placed on knee angle) followed by a vertical jump [9]. Three attempts were allowed with 1 min of passive recovery between them (within-session reliability: ICC = 0.988, *p* < 0.001; CV = 4.3%).

The best performances of the SJ and CMJ tests were used for the subsequent statistical analysis. Flight time was measured using the contact mat and the jump height (cm) was calculated as follows: 9.81 × flight time^2^/8 [12]. In addition, SJ and CMJ jump height and the athletes’ body mass were used to calculate absolute (W) and relative (W·kg^−0.67^) peak power. Absolute peak power was calculated by Equation (1) of Sayers et al. [13]:

Absolute peak power (W) = 60.7 × jump height (cm) + 45.3 × BM (kg) − 2055,(1)

Relative peak power, calculated using allometric scaling, was derived using the following formula: relative peak power (W·kg^−0.67^) = absolute peak power (W)/kg^0.67^ [4,6].

#### 2.4.3. Sport-Specific Anaerobic Performance

*Multiple Frequency Speed of Kick Test (FSKT_mult_)*. The FSKT_mult_ consists of five 10 s sets with a 10 s passive recovery between sets. Each athlete was placed in front of the stand bag equipped with a taekwondo body protector, positioned in same height of the athlete trunk. After the sound signal, athletes executed the maximum number of *bandal-chagi* (i.e., roundhouse kicks) possible, alternating right and left legs. The performance was determined by the number of kicks in each set (n° kicks), total number of kicks (n° kicks), and kick decrement index (KDI) (%) [1,5]. Equation (2) is used to calculate the KDI:

KDI (%) = [1 − (FSKT_1_ + FSKT_2_ + FSKT_3_ + FSKT_4_ + FSKT_5_)/best FSKT × number of sets] × 100, (2)

In addition, in order to express the FSKT_mult_ performance also in terms of power, the method of Tayech et al. [3] was used. Therefore, each athlete was placed in front of the stand bag equipped with a taekwondo body protector, positioned in same height of the athlete trunk at a height (*y*) relative to the mat. During kick execution, the athlete was not to exceed a mark with adhesive tape on the mat, the optimum distance (*x*) determined before the test, to effectively execute kicking. The distances (*x*) and (*y*) allowed for determining the distance (*d*) using the Pythagorean theorem, which is the projection distance of the foot on the body protector. This distance (*d*) allowed for establishing the power of each kick set (see [3,4]). FSKT_mult_ performance were expressed as absolute (W) and relative (W·kg^−0.67^) peak (FSKT_Ppeak_) and mean power (FSKT_Pmean_), and fatigue index (FI) (%). The relative power of each kick set, calculated using allometric scaling (as body mass is a criterion for categorizing taekwondo athletes in competitions) [3,6], was calculated with the following formula: relative power (W·kg^−0.67^) = absolute power (W)/kg^0.67^. Specifically, FSKT_Ppeak_ (W, W·kg^−0.67^): highest power output of the five sets of kicks; FSKT_Pmean_ (W, W·kg^−0.67^): sum of powers of five sets of kicks/5; FI (%): [(FSKT_Ppeak_ − minimum power (FSKT_Pmin_)/FSKT_Ppeak_] × 100.

Tests were recorded, and the videos were analyzed posteriorly to manually count the valid kicks using *Kinovea* software (*version 0.9.5; Joan Charmant and Contributors, Bordeaux, France*) in frame-by-frame mode with an accuracy of 0.03 s. First, the count of a kick started when the athlete moved the attack foot and finished when he/she touched the bag. Kicks considered were those that hit the target during 10 s. If the athlete started the kick before completing 10 s but reached the target only after 10 s, the kick was not taken into account. Second, valid kicks were those performed with appropriate technique and power. To verify the intra-rater reliability of the FSKT_mult_ performance, a researcher (a taekwondo coach, more than 20 years of taekwondo experience and black belt) quantified the valid kicks twice by separating each observation by a 7-day interval [1]. In agreement with the literature [1,5], the ICC (and CV) revealed high reliability with values of 0.988 (0.41%), 0.984 (0.39%), 0.990 (0.20%), 0.994 (0.23%), 0.945 (1.13%), 0.992 (0.37%), and 0.934 (9.01%) (*p* < 0.001) for FSKT_1_, FSKT_2_, FSKT_3_, FSKT_4_, FSKT_5_, FSKT_total_, and KDI, respectively. In addition, a second researcher (a taekwondo coach, more than 30 years of taekwondo experience and black belt) quantified the valid kicks to establish the inter-rater reliability. In agreement with the literature [5], the ICC (and CV) showed high reliability with values of 0.982 (0.64%), 0.967 (0.79%), 0.969 (0.64%), 0.989 (0.55%), 0.924 (1.58%), 0.991 (0.48%), and 0.893 (9.89%) (*p* < 0.001) for FSKT_1_, FSKT_2_, FSKT_3_, FSKT_4_, FSKT_5_, FSKT_total_, and KDI, respectively.

During the FSKT_mult_, HR (b·min^−1^) was measured using a heart rate monitor strap (*Polar H10; Polar Electro Oy, Kempele, Finland*), with an accuracy of 1000 Hz, to quantify mean (HR_mean_) and peak HR (HR_peak_) of the athletes. HR_peak_ was expressed as percentages of the athlete’s theoretical (208 − [0.7 × age]) maximal HR (%HR_max_) [14]. The RPE was recorded immediately after the end of FSKT_mult_ [3,4,6] using the 15-point scale (a.u.), which ranged from 6 (very, very light) to 20 (very, very hard) [15].

### 2.5. Statistical Analysis

Data were analyzed using *IBM SPSS* software (*version 25.0; IBM Corp., Armonk, NY, USA*). Within-session reliability of SJ and CMJ tests measures, as well as for the inter-/intra-rater reliability of the FSKT_mult_, was computed using an average measures two-way random intraclass correlation coefficient (ICC) with absolute agreement and 95% confidence intervals, and the coefficient of variation (CV). The ICC values were interpreted as follows: <0.5: *poor*; 0.5–0.75: *moderate*; 0.75–0.9: *good*; >0.9: *excellent* [16]. CV values ≤ 10% were considered acceptable [17]. The Shapiro–Wilk test revealed the normal distribution of all the considered variables. Therefore, data are presented as mean ± standard deviation [95% confidence interval]. Pearson’s correlation coefficient (*r*) was used to examine relationships between anthropometric and body composition characteristics, muscle power performance (SJ and CMJ), and sport-specific anaerobic performance (FSKT_mult_). The magnitude of correlations was assessed using the following benchmarks: <0.1; *trivial*; 0.1–0.3: *low*; 0.3–0.5: *moderate*; 0.5–0.7: *large*; 0.7–0.9: *very large*; >0.9: *nearly perfect*; =1: *perfect* [18]. Based on the correlation coefficients, simple linear regression was used to model the relationship between a single dependent variable (main FSKT_mult_ performance) with one independent variable (anthropometric and body composition characteristics and muscle power performance). The statistical significance was accepted when *p* < 0.05.

## 3. Results

Anthropometric and body composition characteristics, muscle power performance (SJ and CMJ), and sport-specific anaerobic performance (FSKT_mult_) are provided in Table 1.

Correlations between anthropometric and body composition characteristics, muscle power performance (SJ and CMJ), and sport-specific anaerobic performance (FSKT_mult_) are reported in Table 2, Table 3 and Table 4.

Simple linear regression models to predict main FSKT_mult_ performance (FSKT_total_, relative FSKT_Ppeak_, and relative FSKT_Pmean_) based on anthropometric and body composition characteristics and muscle power performance are reported in Table 5.

## 4. Discussion

The aim of this study was to analyze the relationship between anthropometric and body composition characteristics, muscle power performance (SJ and CMJ), and sport-specific anaerobic performance (FSKT_mult_) in Italian taekwondo athletes.

The FSKT_mult_ is one of the most studied and used sport-specific tests that assesses the anaerobic capacity of taekwondo athletes in intermittent mode [1,5,7,8,9]. Available data indicate that national and international athletes have FSKT_total_ performance between 56 ± 17 and 125 ± 2 kicks [5]. The FSKT_total_ of 86 ± 6 kicks performed by our sample is within this range. Surprisingly, physiological and perceptual measures during the FSKT_mult_ have been neglected in both validation and practical application studies [1,5,7,8,9]. Our study is the first to have quantified the HR (HR_mean_ and HR_peak_) and RPE (Borg 6–20 scale) during the FSKT_mult_ in parallel. The HR_peak_, expressed as %HR_max_ predicted for age [14], was 94 ± 3%, while the RPE was 15 ± 2 a.u. (Table 1). In contrast, the %HR_max_ and RPE of the TAIKT_chest_ were systematically investigated showing values of ~90–93% and ~14 a.u., respectively [3,6,19]. It is important to highlight that typical official match activity elicited near-maximal HR responses (%HR_max_: ~96–97%) and RPE of ~14 a.u. [20,21,22,23,24], in line with our values found in the FSKT_mult_. In addition, we used for the first time the method of Tayech et al. [3], developed for the TAIKT_chest_, to express the performance of the FSKT_mult_ also in terms of absolute and relative power. Both tests have similar methodological and performance characteristics; thus, this method can also be easily applied to the FSKT_mult_ by providing additional performance parameters that consider body mass (i.e., a fundamental criterion for categorizing taekwondo athletes in competitions) [1,3].

The first hypothesis was confirmed as the correlation analysis between muscle power performance and body composition characteristics outlined the pattern of relationship documented earlier [9]. In particular, the SJ and CMJ performance, expressed in terms of height achieved, were significantly and positively correlated with MM (*r* = 0.84 and 0.82, respectively) and negatively with FM (*r* = −0.53 and −0.53, respectively) and BF% (*r* = −0.66 and −0.66, respectively) (Table 2). Similarly, Ojeda-Aravena et al. [9] found that SJ and CMJ performance were significantly and positively correlated with MM (*r* = 0.58) and negatively with FM (*r* = −0.89 and −0.84, respectively) and BF% (*r* = −0.89 and −0.86, respectively) in Chilean taekwondo athletes. The significant and negative relationship between muscle power performance and BF% was previously found in taekwondo [25] as well in other combat sports such as karate and silat [25,26]. In agreement with these studies, we suggest that reducing FM with appropriate physical training and dietary planning supports the improvement of lower limb muscle power [9,25,26]. On the other hand, SJ and CMJ performance, expressed in terms of absolute peak power, were also significantly and positively correlated with BM (and thus also with BMI) but were not correlated with FM and BF%. Equation (1), which is used to calculate the absolute peak power of jumping performance, is based on the achieved vertical height and BM [13]. Therefore, the relationship between these two parameters and their relationship with other body composition characteristics influence the correlation analyses. Consequently, muscle power performance, expressed in terms of peak relative power, showed lower correlation magnitude with BM but higher (and significant) correlation magnitude with BF%, as the allometric scale normalized performance to BM.

The second hypothesis was confirmed as correlations between FSKT_mult_ performance, derived from the number of valid kicks, and body composition characteristics emerged. Specifically, the FSKT_total_ (and most series of the FSKT_mult_) was significantly and positively correlated with MM (*r* = 0.52) and negatively with BF% (*r* = −0.50) (Table 3). Previously, Santos et al. [8] found that only the fourth set of the FSKT_4_ was significantly and negatively correlated with FM (*r* = −0.61) in Brazilian taekwondo athletes. Instead, Ojeda-Aravena et al. [9] reported that FSKT_total_ was significantly and positively correlated with MM (*r* = 0.56). However, it is important to consider that simple linear regression models showed that BF% and MM explained only ~25 and 27% of the variance in FSKT_total_, respectively (Table 5). In line with this, Ojeda-Aravena et al. [9] indicated that MM explained only ~31% of the variance in FSKT_total_. Our results confirm and extend previous ones, supporting the assumption that the optimization of body composition is relevant in taekwondo, as the improvement in ability to repeat intermittent high-intensity efforts depends on MM and its worsening on FM [8,9]. It is interesting to highlight that in our study, only the FSKT_4_ was significantly and positively correlated with BH (*r* = 0.49). In contrast, Santos et al. [8] found that only the FSKT_4_ was significantly and negatively correlated with BH (*r* = −0.51). These contradictory data, when combined with the absence of relationships for other FSKT_mult_ performance, support the independence of FSKT_mult_ from height, as the target is positioned at the same height as the athlete’s trunk. In confirmation, FSKT_mult_ performance, expressed in terms of absolute and relative power, was significantly and positively correlated with BH, as the method of Tayech et al. [3] considers the height of the athlete’s trunk, the optimum distance to effectively execute kicking, and the projection distance of the foot on the body protector. In addition, this method is also based on BM (and lower limb mass) [3], consequently affecting the relationship between absolute and relative power performance of FSKT_mult_ and body composition characteristics.

The third hypothesis was also confirmed as correlations emerged between FSKT_mult_ performance, derived from the number of valid kicks, and muscle power performance, expressed in terms of height achieved. In particular, the FSKT_total_ (and most series of the FSKT_mult_) was significantly and positively correlated with SJ and CMJ performance (*r* = 0.52 and 0.51, respectively) (Table 4). However, SJ and CMJ performance explained only ~27 and 26% of the variance of FSKT_total_, respectively (Table 5). In Brazilian taekwondo athletes, Albuquerque et al. [7] found that FSKT_total_ was significantly and positively correlated with CMJ performance (*r* = 0.44), and the latter explained only ~17% of the variance in FSKT_total_. Taekwondo athletes perform short and dynamic actions, at different joint ranges, generally accelerating limbs until they reach the final range of motion or the opponent [6,10,20]. Therefore, training programs also place emphasis on muscle power [20]. The available data indicate that the ability to repeat high-intensity intermittent efforts in taekwondo depends only in minor part on lower limb power. The specificity of the FSKT_mult_ is based on the alternation of the kick complex motor task [10]. Therefore, the demand of coordination and appropriate technique could impact more on test performance [7]. In addition, during combat, athletes are required to have high precision, but only certain minimum power values, to reach and successfully impact their opponent’s electronic scoring devices [3,6]. It is interesting to highlight that the FSKT_total_ was also significantly and positively correlated with the SJ and CMJ performance normalized by BM with the allometric scale. These results further support the irrelevant influence of BM on muscle power and high-intensity intermittent sport-specific performance found in our study. According to Abidin et al. [25], body size factor does not accurately represent the exact body composition of athletes, highlighting the importance of determining the anthropometric profile and body composition to identify specific characteristics of athletes in a given sport discipline.

### 4.1. Limitations

Although our study offers novel information, several limitations must be acknowledged: First, we used bioelectrical impedance analysis (BIA) to assess body composition as it is recognized as an inexpensive and easy-to-use practical tool [9,27]. However, dual-energy X-ray absorptiometry (DXA) is believed to be a criterion measure for determining body composition, although its use may not be feasible in some laboratory and field testing scenarios due to the cost of the device, radiation exposure, and specific laws for technicians [27,28,29]. Second, athletes were recruited by the same club, were regularly engaged in national and international competitions, and followed the same training program. Nevertheless, variation in athletes’ characteristics in terms of sex and age could generate greater variation in the responses of the variables, highlighting the importance of analyzing these relationships independently as well [9]. Finally, simple linear regression was used to model the relationships and to obtain more stable estimates because the number of subjects, although not underpowered, limited the application of multiple linear regression [7,9].

### 4.2. Practical Applications

Our results could help coaches and strength and conditioning professionals to understand the factors that contribute to or limit the ability to repeat the decisive and dynamic high-intensity actions in taekwondo. Specifically, this study has three main applications: (a) The FSKT_mult_ is a non-invasive and easy to perform sport-specific test, and the %HR_max_ and RPE values recorded in our athletes indicate that it also reproduces typical official match demands [20,21,22,23,24]. In addition, FSKT_mult_ performance can also be easily expressed in terms of absolute and relative power, using the method developed for the TAIKT_chest_ [3], increasing the amount of relevant information that can be derived from the test. In particular, relative FSKT_Ppeak_ and FSKT_Pmean_ performance, normalized by BM with the allometric scale, are useful for comparing athletes of different weight categories. (b) Body composition can be partially changed by targeted training and dietary planning [30]. It should be considered in the interpretation of FSKT_mult_, and for improving the ability to repeat high-intensity intermittent efforts, as athletes with higher MM, and lower BF%, achieve higher FSKT_total_ performance. (c) Lower limb muscle power positively influences the ability to repeat high-intensity intermittent efforts. In this regard, training programs should emphasize ballistic and plyometric exercises [20], as typical kicking techniques are characterized by acceleration of the limbs until the final range of motion, while repeated actions involve eccentric–concentric actions (i.e., the stretch-shortening cycle) [6,10,31].

## 5. Conclusions

The results of the present study suggest that a reduction in fat mass, with appropriate physical training and dietary planning, supports the improvement in lower limb muscle power in taekwondo athletes. In parallel, the optimization of body composition is relevant, as the improvement in ability to repeat intermittent high-intensity efforts depends on muscle mass and its worsening on fat mass. Also, the ability to repeat high-intensity intermittent efforts in taekwondo depends only in minor part on lower limb power, as the demand of coordination and appropriate technique could impact more on test performance. Overall, the present data highlight the existence of a pattern of relationship between the characteristics and physical performance analyzed, which deserves further investigation in the future, and support the use of the FSKT_mult_ in practice to assess the ability to repeat high-intensity intermittent efforts.

## Figures and Tables

**Table 1:** Descriptive statistics of anthropometric and body composition characteristics, muscle power performance (SJ and CMJ), and sport-specific anaerobic performance (FSKT_mult_) in taekwondo athletes (values are presented as mean ± standard deviation [95% confidence interval], N = 19).

Variable	Mean ± SD [95% CI]
**Anthropometric and Body Composition Characteristics**	
BH (cm)	171.16 ± 4.90 [168.80–173.52]
BM (kg)	58.02 ± 6.61 [54.84–61.21]
BMI (kg·m^−2^)	19.79 ± 1.90 [18.88–20.71]
FM (kg)	8.91 ± 5.70 [6.16–11.65]
BF (%)	15.06 ± 8.38 [11.02–19.10]
MM (kg)	46.64 ± 5.80 [43.84–49.43]
LBM (kg)	49.12 ± 6.09 [46.18–52.05]
**Muscle Power Performance**	
SJ_height_ (cm)	29.0 ± 6.9 [25.6–32.3]
SJ_Ppeak_ (W)	2331.96 ± 589.06 [2048.04–2615.88]
SJ_Ppeak_ (W·kg^−0.67^)	152.45 ± 31.41 [137.31–167.59]
CMJ_height_ (cm)	31.9 ± 7.3 [28.4–35.4]
CMJ_Ppeak_ (W)	2490.00 ± 596.35 [2202.57–2777.44]
CMJ_Ppeak_ (W·kg^−0.67^)	164.32 ± 32.16 [148.82–179.82]
**Sport-Specific Anaerobic Performance**	
FSKT_1_ (n° kicks)	18 ± 1 [18–19]
FSKT_2_ (n° kicks)	18 ± 1 [18–19]
FSKT_3_ (n° kicks)	17 ± 1 [17–18]
FSKT_4_ (n° kicks)	16 ± 1 [15–17]
FSKT_5_ (n° kicks)	16 ± 1 [15–16]
FSKT_total_ (n° kicks)	86 ± 6 [83–89]
KDI (%)	7 ± 3 [6–9]
FSKT_Ppeak_ (W)	6.92 ± 1.54 [6.18–7.66]
FSKT_Ppeak_ (W·kg^−0.67^)	0.45 ± 0.08 [0.41–0.49]
FSKT_Pmean_ (W)	5.98 ± 1.27 [5.37–6.60]
FSKT_Pmean_ (W·kg^−0.67^)	0.39 ± 0.07 [0.36–0.42]
FI (%)	28 ± 10 [23–33]
HR_mean_ (b·min^−1^)	176 ± 9 [172–180]
HR_peak_ (b·min^−1^)	184 ± 7 [181–188]
%HR_max_	94 ± 3 [93–95]
RPE (a.u.)	15 ± 2 [15–16]

Notes: BH: body height; BM: body mass; BMI: body mass index; FM: fat mass; BF: body fat; MM: muscle mass; LMB: lean body mass; SJ: squat jump; CMJ: countermovement jump; P_peak_: peak power; FSKT_1_: set 1; FSKT_2_: set 2; FSKT_3_: set 3; FSKT_4_: set 4; FSKT_5_: set 5; FSKT_total_: total number of kicks in the 5 sets; KDI: kick decrement index; P_mean_: mean power; FI: fatigue index; HR_mean_: mean heart rate; HR_peak_: peak heart rate; %HR_max_: percentages of the athlete’s theoretical maximal HR; RPE: rating of perceived exertion (Borg 6–20 scale) scale.

**Table 2:** Correlation coefficients (*r*) between anthropometric and body composition characteristics and muscle power performance (SJ and CMJ) in taekwondo athletes (N = 19).

		Anthropometric and Body Composition Characteristics						
		BH (cm)	BM (kg)	BMI (kg·m^−2^)	FM (kg)	BF (%)	MM (kg)	LBM (kg)
**Muscle Power Performance**	SJ_height_ (cm)	0.546 *	0.326	0.074	−0.525 *	−0.661 **	0.844 **	0.845 **
	SJ_Ppeak_ (W)	0.649 **	0.740 **	0.496 *	−0.110	−0.305	0.905 **	0.906 **
	SJ_Ppeak_ (W·kg^−0.67^)	0.630 **	0.554 *	0.292	−0.322	−0.497 *	0.901 **	0.902 **
	CMJ_height_ (cm)	0.553 *	0.297	0.036	−0.530 *	−0.659 **	0.817 **	0.818 **
	CMJ_Ppeak_ (W)	0.644 **	0.728 **	0.485 *	−0.105	−0.294	0.887 **	0.888 **
	CMJ_Ppeak_ (W·kg^−0.67^)	0.633 **	0.503 *	0.231	−0.354	−0.519 *	0.876 **	0.877 **

Notes: BH: body height; BM: body mass; BMI: body mass index; FM: fat mass; BF: body fat; MM: muscle mass; LMB: lean body mass; SJ: squat jump; CMJ: countermovement jump; P_peak_: peak power. * *p* < 0.05, ** *p* < 0.01.

**Table 3:** Correlation coefficients (*r*) between anthropometric and body composition characteristics, and sport-specific anaerobic performance (FSKT_mult_) in taekwondo athletes (N = 19).

		Anthropometric and Body Composition Characteristics						
		BH (cm)	BM (kg)	BMI (kg·m^−2^)	FM (kg)	BF (%)	MM (kg)	LBM (kg)
**Sport-Specific Anaerobic** **Performance**	FSKT_1_ (n° kicks)	0.334	0.111	−0.050	−0.404	−0.481 *	0.498 *	0.499 *
	FSKT_2_ (n° kicks)	0.348	0.145	−0.016	−0.395	−0.490 *	0.526 *	0.527 *
	FSKT_3_ (n° kicks)	0.363	0.008	−0.183	−0.423	−0.479 *	0.403	0.404
	FSKT_4_ (n° kicks)	0.490 *	0.205	−0.035	−0.336	−0.447	0.536 *	0.537 *
	FSKT_5_ (n° kicks)	0.337	0.110	−0.054	−0.267	−0.356	0.368	0.369
	FSKT_total_ (n° kicks)	0.416	0.133	−0.070	−0.403	−0.499 *	0.521 *	0.522 *
	KDI (%)	−0.167	−0.020	0.073	−0.050	−0.039	0.026	0.025
	FSKT_Ppeak_ (W)	0.708 **	0.737 **	0.461 *	0.199	0.030	0.613 **	0.613 **
	FSKT_Ppeak_ (W·kg^−0.67^)	0.685 **	0.489 *	0.188	0.014	−0.116	0.517 *	0.517 *
	FSKT_Pmean_ (W)	0.745 **	0.768 **	0.475 *	0.256	0.083	0.593 **	0.593 **
	FSKT_Pmean_ (W·kg^−0.67^)	0.739 **	0.524 *	0.198	0.073	−0.063	0.500 *	0.501 *
	FI (%)	−0.148	−0.015	0.074	−0.084	−0.057	0.063	0.062

Notes: BH: body height; BM: body mass; BMI: body mass index; FM: fat mass; BF: body fat; MM: muscle mass; LMB: lean body mass; FSKT_1_: set 1; FSKT_2_: set 2; FSKT_3_: set 3; FSKT_4_: set 4; FSKT_5_: set 5; FSKT_total_: total number of kicks in the 5 sets; KDI: kick decrement index; P_peak_: peak power; P_mean_: mean power; FI: fatigue index. * *p* < 0.05, ** *p* < 0.01.

**Table 4:** Correlation coefficients (*r*) between muscle power performance (SJ and CMJ) and sport-specific anaerobic performance (FSKT_mult_) in taekwondo athletes (N = 19).

		Muscle Power Performance					
		SJ_height_ (cm)	SJ_Ppeak_ (W)	SJ_Ppeak_ (W·kg^−0.67^)	CMJ_height_ (cm)	CMJ_Ppeak_ (W)	CMJ_Ppeak_ (W·kg^−0.67^)
**Sport-Specific Anaerobic** **Performance**	FSKT_1_ (n° kicks)	0.443	0.372	0.424	0.468 *	0.422	0.452
	FSKT_2_ (n° kicks)	0.559 *	0.471 *	0.548 *	0.564 *	0.490 *	0.560 *
	FSKT_3_ (n° kicks)	0.484 *	0.348	0.445	0.482 *	0.363	0.454
	FSKT_4_ (n° kicks)	0.560 *	0.503 *	0.567 *	0.542 *	0.477 *	0.554 *
	FSKT_5_ (n° kicks)	0.294	0.265	0.318	0.224	0.211	0.255
	FSKT_total_ (n° kicks)	0.520 *	0.438	0.512 *	0.508 *	0.439	0.507 *
	KDI (%)	0.089	0.053	0.051	0.169	0.124	0.130
	FSKT_Ppeak_ (W)	0.440	0.687 **	0.589 **	0.435	0.708 **	0.573 *
	FSKT_Ppeak_ (W·kg^−0.67^)	0.419	0.547 *	0.508 *	0.427	0.577 **	0.512 *
	FSKT_Pmean_ (W)	0.397	0.672 **	0.566 *	0.368	0.672 **	0.526 *
	FSKT_Pmean_ (W·kg^−0.67^)	0.377	0.534 *	0.489 *	0.355	0.539 *	0.463 *
	FI (%)	0.125	0.081	0.083	0.190	0.155	0.150

Notes: SJ: squat jump; CMJ: countermovement jump; P_peak_: peak power; FSKT_1_: set 1; FSKT_2_: set 2; FSKT_3_: set 3; FSKT_4_: set 4; FSKT_5_: set 5; FSKT_total_: total number of kicks in the 5 sets; KDI: kick decrement index; P_mean_: mean power; FI: fatigue index. * *p* < 0.05, ** *p* < 0.01.

**Table 5:** Simple linear regression models to estimate main FSKT_mult_ performance from anthropometric and body composition characteristics and muscle power performance (SJ and CMJ) in taekwondo athletes (N = 19).

	Equations	*R* ^2^	Adjusted *R*^2^	SEE	*p*
**FSKT_total_** **(n° kicks)**	91.33 − 0.36 (BF%)	0.25	0.21	5.40	0.030
	60.56 + 0.54 (MMkg)	0.27	0.23	5.32	0.022
	60.44 + 0.52 (LBMkg)	0.27	0.23	5.32	0.022
	72.67 + 0.46 (SJ_height_cm)	0.27	0.23	5.32	0.022
	70.84 + 0.10 (SJ_PpeakW_·kg^−0.67^)	0.26	0.22	5.35	0.025
	72.38 + 0.42 (CMJ_height_cm)	0.26	0.22	5.37	0.026
	70.20 + 0.10 (CMJ_Ppeak_W·kg^−0.67^)	0.26	0.21	5.37	0.027
**FSKT_Ppeak_** **(W·kg^−0.67^)**	–12.02 + 0.09 (BHcm)	0.47	0.43	0.49	0.001
	0.81 + 0.05 (BMkg)	0.23	0.20	0.59	0.033
	0.91 + 0.06 (MMkg)	0.27	0.22	0.58	0.023
	0.90 + 0.06 (LBMkg)	0.27	0.23	0.58	0.023
	2.21 + 0.001 (SJ_Ppeak_W)	0.30	0.26	0.56	0.015
	2.01 + 0.01 (SJ_Ppeak_W·kg^−0.67^)	0.26	0.22	0.58	0.026
	2.05 + 0.001 (CMJ_Ppeak_W)	0.33	0.29	0.55	0.010
	1.91 + 0.01 (CMJ_Ppeak_W·kg^−0.67^)	0.26	0.22	0.58	0.025
**FSKT_Pmean_** **(W·kg^−0.67^)**	–10.64 + 0.08 (BHcm)	0.55	0.52	0.37	0.0003
	0.68 + 0.04 (BMkg)	0.28	0.23	0.47	0.021
	0.99 + 0.05 (MMkg)	0.25	0.21	0.48	0.029
	0.98 + 0.04 (LBMkg)	0.25	0.21	0.48	0.029
	2.01 + 0.0005 (SJ_Ppeak_W)	0.29	0.24	0.46	0.018
	1.87 + 0.01 (SJ_Ppeak_W·kg^−0.67^)	0.24	0.20	0.48	0.033
	1.93 + 0.0004 (CMJ_Ppeak_W)	0.29	0.25	0.46	0.017
	1.87 + 0.01 (CMJ_Ppeak_W·kg^−0.67^)	0.21	0.17	0.49	0.046

Notes: *R*^2^: coefficient of determination value; SEE: standard error of the estimate; *p* = significance level; FSKT_total_: total number of kicks in the 5 sets; P_peak_: peak power; P_mean_: mean power; BF: body fat; MM: muscle mass; LMB: lean body mass; SJ: squat jump; CMJ: countermovement jump; BH: body height; BM: body mass.

## Data Availability

Data generated or analyzed during this study are available from the corresponding author upon reasonable request.
